# Predicting Early Cochlear Implant Performance: Can Cognitive Testing Help?

**DOI:** 10.1097/ONO.0000000000000050

**Published:** 2024-03-21

**Authors:** Natalie Schauwecker, Terrin N. Tamati, Aaron C. Moberly

**Affiliations:** 1 Department of Otolaryngology – Head and Neck Surgery, Vanderbilt University Medical Center, Nashville, Tennessee; 2 Department of Otorhinolaryngology/Head and Neck Surgery, University of Groningen, University Medical Center Groningen, Groningen, The Netherlands.

**Keywords:** Cochlear implant, Cognition, Outcomes, Speech recognition

## Abstract

**Introduction:**

There is significant variability in speech recognition outcomes in adults who receive cochlear implants (CIs). Little is known regarding cognitive influences on very early CI performance, during which significant neural plasticity occurs.

**Methods:**

Prospective study of 15 postlingually deafened adult CI candidates tested preoperatively with a battery of cognitive assessments. The mini-mental state exam (MMSE), forward digit span, Stroop measure of inhibition-concentration, and test of word reading efficiency were utilized to assess cognition. consonant-nucleus-consonant words, AZBio sentences in quiet, and AZBio sentences in noise (+10 dB SNR) were utilized to assess speech recognition at 1- and 3-months of CI use.

**Results:**

Performance in all speech measures at 1-month was moderately correlated with preoperative MMSE, but these correlations were not strongly correlated after correcting for multiple comparisons. There were large correlations of forward digit span with 1-month AzBio quiet (*P* ≤ 0.001, rho = 0.762) and AzBio noise (*P* ≤ 0.001, rho = 0.860), both of which were strong after correction. At 3 months, forward digit span was strongly predictive of AzBio noise (*P* ≤ 0.001, rho = 0.786), which was strongly correlated after correction. Changes in speech recognition scores were not correlated with preoperative cognitive test scores.

**Conclusions:**

Working memory capacity significantly predicted early CI sentence recognition performance in our small cohort, while other cognitive functions assessed did not. These results differ from prior studies predicting longer-term outcomes. Findings and further studies may lead to better preoperative counseling and help identify patients who require closer evaluation to ensure optimal CI performance.

For the last forty years, cochlear implantation has provided restoration of audibility in adults and children with otherwise unserviceable sensorineural hearing loss (1,2). Cochlear implants (CIs) directly stimulate the auditory nerve, providing auditory input to the user (3). Despite advancements in CI technology and surgical techniques, CI outcomes have remained remarkably variable, with reports of up to 50% of users unable to use the telephone and more than 10% unable to correctly recognize greater than 10% of words in quiet (4,5). Initially, studies on CI outcome variability assessed factors affecting the signal delivered from the implant to the auditory nerve, including the design of the electrode as well as surgical techniques (6,7). Other studies have investigated factors related to input from the CI and processing from the auditory nerve to the auditory cortex, as well as the role of duration of deafness and patient age on performance (8). Despite these investigations, much of the variability in performance cannot be predicted by standard clinical and demographic characteristics alone (5,9).

More recently, cognitive function has been assessed and found to contribute significantly to speech recognition outcomes in experienced CI users (10–12). CIs supply patients with spectrotemporally degraded speech signals when compared to acoustic hearing. As a result, it is hypothesized that CI recipients are required to utilize cognitive resources to make sense of the new electrical sound input (13). There is a large volume of research on the contributions of cognitive functions to speech processing under adverse listening situations (14–16). Due to the degraded signal provided, CI-listening can be considered a form of adverse listening. Prior studies have demonstrated associations between speech recognition outcomes and working memory, phonological processing speed, and inhibition-concentration measured cross-sectionally in experienced CI users (8,12).

Fewer studies have examined preoperative cognitive functions as longitudinal predictors of postoperative speech recognition outcomes in CI users. Zhan et al (4) demonstrated that inhibition-concentration measured preoperatively significantly predicted CI speech recognition performance at 6 months postoperatively. Similarly, Moberly et al (13) demonstrated that a preoperative non-word reading efficiency predicted 6-month postoperative sentence recognition in new adult CI users. However, there has been limited investigation in early (1- and 3-months) speech recognition performance and cognitive abilities in new CI users. Importantly, it has been demonstrated in hearing aid users that as one becomes accustomed to the altered auditory input, cognitive functions may influence performance less, suggesting that the role of cognition in speech recognition may change over time while adjusting to the listening device (14). This study sought to investigate the contribution of baseline cognitive abilities before implantation to early speech recognition performance, as well as changes in speech recognition performance from preoperative performance, at 1- and 3-months post-CI activation. We predicted that early in the first few months after implantation, while patients adjust to the degraded speech input, cognitive functions would predict CI performance differently, perhaps even more strongly, when compared with prior investigations of 6- and 12-month CI performance. Multiple clinical speech recognition measures were tested including words, sentences in quiet, and sentences in multitalker babble (noise). Our hypothesis was that cognition would predict sentence and word recognition early after implantation.

## METHODS

### Participants

Fifteen postlingually deafened, adult CI candidates (8 males, 7 females), were recruited from September 2018 to December 2020 from a single large-volume tertiary neurotology practice. Patients were assessed preoperatively, as well as at the 1- and 3-month mark post-CI activation. Preoperative cognitive assessments were completed within 2 months before the implantation date. These assessments took approximately 90 minutes to complete. All candidates were native American English speakers who obtained at least some high school education. Socioeconomic status (SES) was quantified based on occupational and educational levels, each defined on a scale from 1 (lowest) to 8 (highest), with these 2 scores multiplied, providing a score between 1 and 64. Two participants reported their occupation only as “retired,” so SES was not computed for these 2 participants (17). No candidates had prior evidence of dementia, nor stroke or seizure history. All patients received Cochlear Americas CIs and were implanted by one of the center’s 3 neurotologists. No participants had a history of prior cochlear implantation. Fourteen participants received a single CI, and 1 received bilateral simultaneous CIs. Neither the electrode array used nor datalogging data were available for the study participants. Demographics, including age, gender, and hearing history were collected. Details are enumerated in Table 1. Participants gave informed written consent for study participation, which was approved by the local Institutional Review Board.

**TABLE 1.** Candidate demographics and hearing history

Candidate	Gender	Age (years)	SES	HL Etiology	Duration HL (years)	Better-ear PTA	Best-aided condition
1	M	55	42	Idiopathic	42	80	Bimodal—L HA, R CI
2	M	74	36	Noise exposure	44	91.25	Bimodal—L HA, R CI
3	F	61	–	Noise exposure	41	73.75	Bimodal—L HA, R CI
4	F	58	15	Genetic, progressive as adult	52	120	Bilateral CI
5	M	68	42	Noise exposure	15	71	Bimodal—L HA, R CI
6	M	75	15	Noise exposure	35	76	Bimodal—L HA, R CI
7	F	54	42	Genetic	53.5	106.25	Bimodal—L HA, R CI
8	F	75	39	Idiopathic, progressive as adult	18	67	Bimodal—L HA, R CI
9	M	67	49	Genetic	22	78	Bimodal—L HA, R CI
10	M	65	9	Meniere’s	40	65	Bimodal—L CI, R HA
11	M	65	20	Noise exposure	15	80	Bimodal—L CI, R HA
12	F	53	30	Genetic, progressive as adult	41	80	Bimodal—L HA, R CI
13	M	73	32	Genetic, progressive as adult	33	66	Bimodal—L HA, R CI
14	F	49	42	Genetic, progressive as adult	49	102	Bimodal—L HA, R CI
15	F	76	-	Idiopathic	26	88	Bimodal—L HA, R CI

CI indicates cochlear implant; F, female; HA, hearing aid; HL, hearing loss; L, left ear; M, male; PTA, unaided pure tone average in dB HL; R, right ear; SES, socioeconomic status.

### Cognitive Testing

All participants underwent a battery of preoperative cognitive tests, which have been applied extensively in CI users and have been found to be useful in uncovering the relationship between cognitive function and individual differences in speech recognition outcomes in experienced CI users. The measures consisted of the mini-mental state exam (MMSE), forward digit span, a test of word reading efficiency, and the Stroop task (4,8,11–13).

The MMSE (18) is the best-known and most frequently utilized tool to screen for early signs of cognitive impairment (17). It assesses 6 areas of cognitive abilities including orientation, attention and concentration, recall, language, visuospatial abilities, and abilities to follow complex commands (16). Participants read the instructions and were provided visual stimuli to complete this exam to avoid any poor audibility effects. A score less than 26 is concerning for possible cognitive decline, although some studies suggest that a cutoff of 24 is more specific for detecting dementia (19).

Working memory capacity was tested using a visual forward digit span task based on the Wechsler Intelligence Scale for Children (20). Visual sequences of 2 to 9 digits were presented via computer screen, 1 digit at a time to participants. Visual cues were used to eliminate the effects of poor audibility on participant performance. Participants were then required to “repeat” back the digit sequence by touching the correct digits in order on the computer screen. Before testing participants were familiarized with the task and allowed to practice. Participants were encouraged to guess if unsure. Performance was determined by the total proportion of correctly recalled digits. No feedback was provided during the task itself.

A test of real-word and non-word reading efficiency was used to assess lexical and phonological processing (21). The Test of Word Reading Efficiency, Second Edition (TOWRE-2) was administered visually to participants (13). The initial subtest assessed sight word efficiency (SWE), where candidates read aloud as many real words as possible from a preset list. The SWE raw score was determined by the total number of correct real words. The final subtest assessed phonological decoding efficiency (PDE), where candidates read aloud as many nonsensical words as possible from a preset list. The PDE raw score was determined by the total number of correct non-words.

The Stroop task, including both congruent and incongruent trials, assesses concentration and inhibitory control respectively. This study utilized a computer-based version of the Stroop-Color-Word Interference Test (22). This task analyzes how quickly candidates can identify the correct ink color depicted by a color word label. In the congruent trial, the ink color and color word matches, while in the incongruent trial, the ink color and color word do not match. Participant response times to each of these conditions were computed and used as the metrics of performance.

### Audiometric Analysis

All candidates underwent 1- and 3-month postoperative speech recognition testing. Consonant-Nucleus-Consonant (CNC) words, AzBio sentences in quiet (AzBio quiet), and AzBio sentences in multitalker babble at +10 dB SNR (AzBio noise) in the best-aided condition were assessed for each participant (23,24). The best-aided condition was defined as the everyday listening condition for that participant. Thus, bimodal participants (those listening with a CI and a hearing aid in the contralateral ear) were tested in the bimodal condition, which comprised 14 out of 15 participants. The single simultaneous bilateral CI patient was tested in bilateral CI condition. Individual patients’ best-aided conditions are listed in Table 1. Clinical preoperative CNC, AzBio quiet, and AzBio noise scores were obtained from the participants’ electronic medical records, where available. Participants completed preoperative and postoperative speech recognition testing in their best-aided listening configurations.

### Data Analysis

The Statistical Package for Social Science (SPSS) (25) was utilized to test the hypothesis that preoperative cognitive functioning would predict early postoperative speech recognition performance. Spearman rho correlations were used considering speech recognition performance scores are not normally distributed. Correlations were assessed among preoperative cognitive tasks and both 1- and 3-month speech performance metrics. Correlations were also assessed for the change (delta) in speech recognition from preoperative to 1- and 3-months postoperative performance for CNC and AzBio quiet. Delta scores were not computed for AzBio noise, as only 7 participants had preoperative AzBio noise scores. Additionally, patient factors, including age, duration of hearing loss (computed as age minus reported age at onset of hearing loss), and baseline preoperative hearing were analyzed to assess for effects on early CI performance. Spearman correlation coefficients are only reported where *P* < 0.05. However, Holm-Bonferroni corrections were then applied to correct for multiple comparisons. Uncorrected *P* values are reported, but results are reported as significant only where surviving Holm-Bonferroni correction. Effect sizes for Spearman rho are as follows: rho = 0.10, small; rho = 0.30, medium; rho = 0.50, large effect (26).

## RESULTS

All participants had preoperative CNC testing and preoperative AzBio quiet, and 47% had AzBio noise scores from the clinic before surgery. All participants underwent MMSE, digit span, TOWRE, and Stroop testing preoperatively. However, 1 patient did not complete all components of Stroop tasks and that participant’s Stroop scores were not included. Table 2 demonstrates preoperative group speech recognition and cognitive testing.

**TABLE 2.** Preoperative speech recognition and cognitive testing

Preoperative testing	Average	N	SD
CNC (% correct)	19.2	15	22.23
AzBio quiet (% correct)	27.8	15	25.59
AzBio noise (% correct)	27.6	7	20.65
MMSE (score)	28.2	15	1.78
Forward digit span (total correct)	40.8	15	22.28
TOWRE-2 (sight word efficiency, SWE)	77.5	15	11.7
TOWRE-2 (phonological decoding efficiency, PDE)	38.6	15	12.9
Stroop congruent (ms)	1350.6	15	333.8
Stroop incongruent (ms)	1601.3	14	386.9

N indicates number of participants; SD, standard deviation of score.

All candidates demonstrated improvement in at least 1 modality of speech recognition from preoperatively to 1- or 3-month testing. At 1-month, CNC scores on average improved by 17% (range −24%–66%, n = 15), AzBio quiet by 30% (range −1%–73%, n = 14), and AzBio noise by 16% (range −4%–46%, n = 7). At 3 months, CNC scores on average improved by 40% (range 8%–68%, n = 15), AzBio quiet by 46% (range −4–91%, n = 13), and AzBio noise by 33% (range 8–66, n = 7).

To further characterize our data, the relations among preoperative cognitive measures are shown in Table 3. Cognitive measures were not strongly correlated, except for the 2 Stroop measures. The relations between preoperative cognitive measures, postoperative speech recognition performance, and preoperative demographic/audiologic measures are shown in Tables 4 and 5. Few moderate to strong correlations of demographics were found with preoperative cognitive test scores. Age was only weakly to moderately correlated with preoperative cognitive measures, while duration of hearing loss and preoperative better-ear unaided PTA were strongly related to one or both Stroop scores. Demographic factors did not predict 1- or 3-month speech recognition test scores.

**TABLE 3.** Spearman rank correlation coefficients (rho) among preoperative cognitive tests

Cognitive test	MMSE	Forward digit span	TOWRE-2 SWE	TOWRE-2 PDE	Stroop congruent	Stroop incongruent
MMSE (score)	1					
Forward digit span (total correct)	0.43	1				
TOWRE-2 (sight word efficiency, SWE)	−0.19	0.02	1			
TOWRE-2 (phonological decoding efficiency, PDE)	0.49	0.44	0.27	1		
Stroop congruent (ms)	0.13	0.28	0.18	0.31	1	
Stroop incongruent (ms)	0.23	0.19	0.22	0.47	0.90	1

**TABLE 4.** Spearman rank correlation coefficients (rho) between preoperative cognitive tests and preoperative demographic/audiologic measures as well as postoperative speech recognition testing

Cognitive test	Age	Duration of hearing loss (years)	Better-ear unaided pure tone average (dB HL)
MMSE (score)	−0.04	−0.11	−0.16
Forward digit span (total correct)	0.10	0.03	−0.28
TOWRE-2 (sight word efficiency, SWE)	0.49	−0.44	−0.33
TOWRE-2 (phonological decoding efficiency, PDE)	−0.02	−0.34	−0.46
Stroop congruent (ms)	0.41	−0.66	−0.49
Stroop incongruent (ms)	0.45	−0.63	−0.58
**Postoperative speech recognition**	**Age**	**Duration of hearing loss (years)**	**Better-ear unaided pure tone average (dB HL)**
1-month postoperative			
CNC (% words correct)	0.27	−0.21	−0.06
AzBio quiet sentences (% words correct)	0.09	−0.20	−0.36
AzBio noise sentences (% words correct)	0.27	−0.16	−0.37
3 months postoperative			
CNC (% words correct)	0.12	−0.24	−0.20
AzBio quiet sentences (% words correct)	0.09	−0.26	−0.18
AzBio noise sentences (% words correct)	0.14	0.07	−0.22

**TABLE 5.** Spearman rank correlation coefficients (rho) among preoperative cognitive tests and postoperative speech recognition change scores

Postoperative speech recognition	MMSE	Forward digit span	TOWRE-2 SWE	TOWRE-2 PDE	Stroop congruent	Stroop incongruent
1-month postoperative						
CNC change (%, 1-month score minus pre-op score)	0.25	0.12	−0.17	−0.18	−0.01	−0.34
AzBio quiet change (%, 1-month score minus pre-op score)	−0.02	0.11	0.06	−0.07	0.03	−0.16
3 months postoperative						
CNC change (%, 3-month score minus pre-op score)	−0.37	−0.42	0.18	−0.41	−0.34	−0.41
AzBio quiet change (%, 3-month score minus pre-op score)	−0.38	−0.42	0.26	−0.27	−0.12	−0.31

**FIG. 1.**
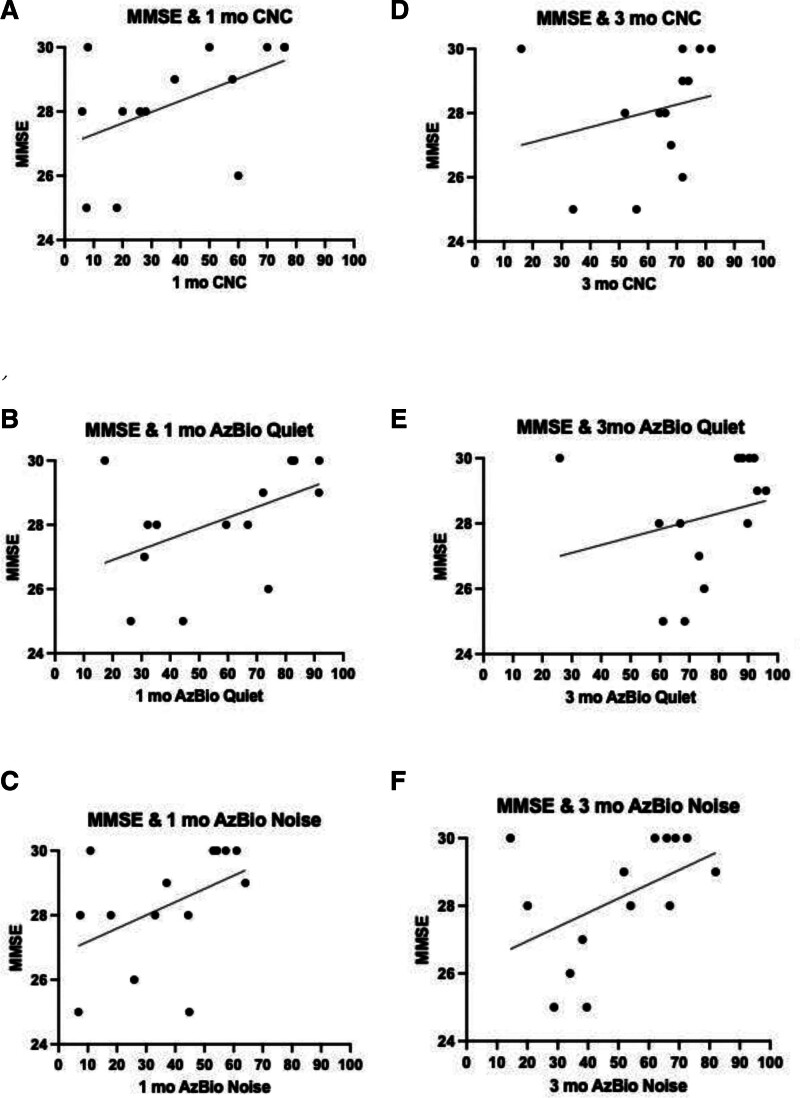
Preoperative mini-mental state exam (MMSE) scores and correlations with 1-month speech recognition testing. *A,* MMSE and 1-month CNC performance; *P* = 0.035, rho = 0.566. *B,* MMSE and 1-month AzBio quiet performance; *P* = 0.041, rho = 0.531. *C,* MMSE and 1-month AzBio noise performance; *P* = 0.045, rho = 0.543. *D,* MMSE and 3-month CNC performance; *P* = 0.104, rho = 0.472. *E,* MMSE and 3-month AzBio quiet performance; *P* = 0.164, rho = 0.394; *F,* MMSE and 3-month AzBio noise performance; *P* = 0.095, rho = 0.464.

In terms of our main analyses, preoperative MMSE scores were evaluated for their association with 1-month speech recognition performance. There was a moderate correlation between MMSE scores and 1-month CNC words (*P* = 0.035, rho = 0.566), AzBio quiet (*P* = 0.041, rho = 0.531), and AzBio noise (*P* = 0.045, rho = 0.543); however, none of these relations were significant after Holm-Bonferroni correction (Fig. 1). Preoperative MMSE scores were not strongly correlated with any 3-month speech recognition scores, nor were they correlated with the delta in speech recognition scores from preoperative to 1- or 3-month testing.

**FIG. 2.**
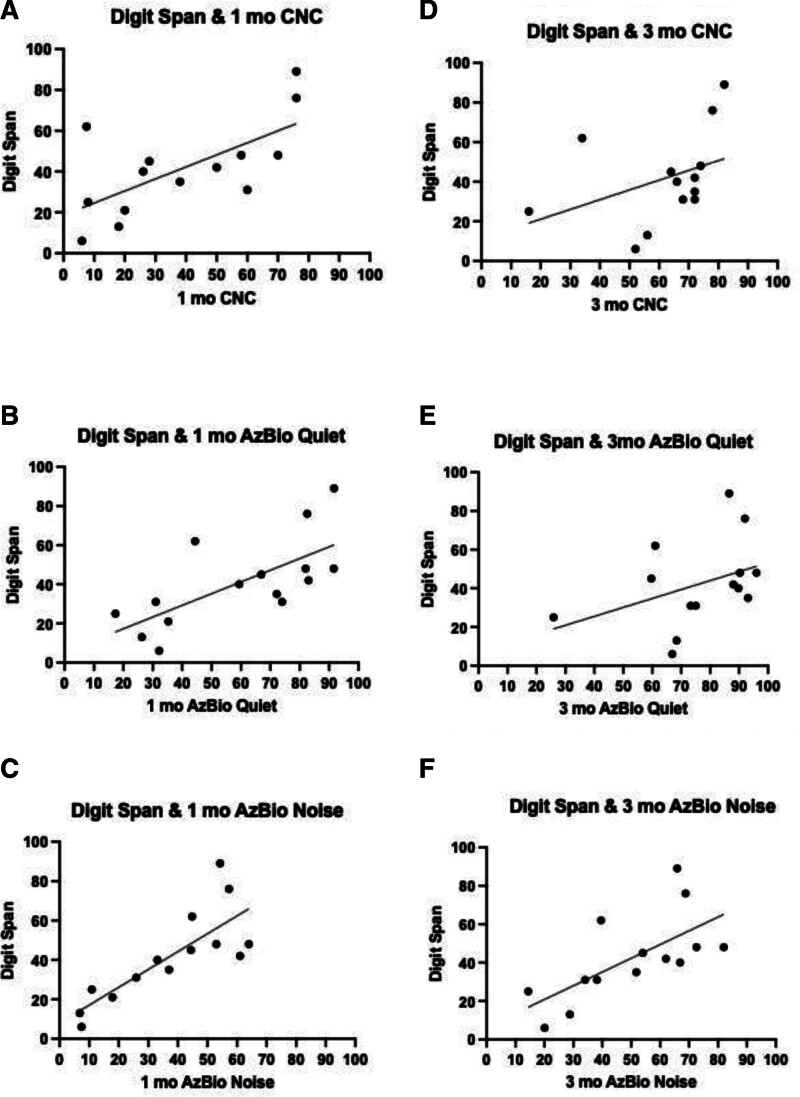
Preoperative digit span scores and correlations with 1- and 3-month speech recognition performance. *A,* Digit span and 1-month CNC performance; *P* ≤ 0.001, rho = 0.762. *B,* Digit span and 1-month AzBio quiet performance; *P* ≤ 0.001, rho = 0.860. *C,* Digit span and 1-month AzBio noise performance; *P* = 0.012, rho = 0.652. *D,* Digit span and 3-month CNC performance; *P* = 0.033, rho = 0.592. *E,* Digit span and 3-month AzBio quiet performance; *P* = 0.148, rho = 0.407. *F,* Digit span and 3-month AzBio noise performance; *P* ≤ 0.001, rho = 0.786.

Next, forward digit span performance was evaluated for its association with 1-month speech recognition testing. There was a moderate correlation for CNC words (*P* = 0.012, rho = 0.652), but this was not significant after Holm-Bonferroni correction. There were large correlations of digit span with AzBio quiet (*P* ≤ 0.001, rho = 0.762) and AzBio noise (*P* ≤ 0.001, rho = 0.860), both of which remained strong after Holm-Bonferroni correction (Fig. 2). At 3-month testing, Digit Span was strongly predictive of AzBio noise (*P* ≤ 0.001, rho = 0.786), which was significant after Holm-Bonferroni correction, and moderately predictive of CNC words (*P* = 0.033, rho = 0.592), but this was not the case after Holm-Bonferroni correction (Fig. 2). Digit span was not correlated with AzBio quiet performance at 3 months (*P* = 0.148). Stroop performance scores and TOWRE-2 reading efficiency scores were not significantly correlated with any scores of 1- or 3- month speech recognition performance, nor were they correlated with the delta in speech recognition scores from preoperative to 1- or 3-month testing.

## DISCUSSION

The purpose of this study was to determine whether preoperative cognitive testing could predict early (1- and 3-month) CI outcomes. It is known that “top-down” cognitive processes are essential when “bottom-up” sensory input is degraded (11). Prior studies have shown that cognitive functions assessed both pre- and post-operatively correlate with longer-term outcomes in experienced CI users at 6 months and beyond postimplantation, with better inhibitory control, concentration, and lexical and phonological processing predicting better outcomes (4,11,13). Our group has also shown correlations of working memory with long-term CI performance (22). Additionally, prior studies have described up to 1–2 years for peak performance with a CI (27–29). It is known that soon after CI activation, the new signals received via the implant provide degraded sensory input to the listener. We expect to see preexisting cognitive functions impact CI performance perhaps even more strongly during this early postactivation period as the brain is effortfully processing the degraded speech input. Out of a battery of preoperative cognitive testing, we found that performance on the forward digit span task was correlated with higher early CI performance on some speech recognition tasks in our small cohort. MMSE scores also correlated with higher early CI performance, but these relations did not survive Holm-Bonferroni correction for multiple comparisons.

Our visual forward digit span task assesses working memory capacity. Working memory is defined as a limited capacity cognitive system, responsible for the temporary holding of information for further processing (30). Working memory capacity is known to play a vital role in recognizing and comprehending spoken language. Prior studies have demonstrated the role of working memory in adults with hearing loss and CI users alike in speech recognition tasks with a degraded auditory signal (11,22,31,32). Therefore, it was not surprising to see that participants with robust working memory displayed higher speech recognition performance on AzBio sentences in quiet and AzBio sentences in noise at 1-month. We did find it interesting that forward digit span was not strongly correlated with AzBio quiet scores at 3 months, but remained strongly correlated for AzBio in noise testing. One could hypothesize that by 3 months CI users have adapted to the degraded auditory input from the implant, and only in more challenging conditions like speech-in-noise are users still required to heavily rely on working memory.

The MMSE broadly assesses multiple areas of cognition, acting as a screening tool for patients who may warrant further cognitive evaluation. MMSE scores showed moderate correlations with 1-month speech recognition outcomes, but these did not survive Holm-Bonferroni correction, possibly based on our small sample size. MMSE scores were not at all predictive of speech recognition at the 3-month mark. There is a well-known association of hearing loss with cognitive decline, with the MMSE as the most common test to screen for deficits in cognitive function (33,30). However, our study, and to the best of our knowledge, those before it, do not divide the MMSE into specific areas of cognitive ability to assess which, if any, correlates specifically, with hearing loss, or ultimately CI performance. In our study, the MMSE should be considered a cognitive screening tool, and, if patients perform poorly, this warrants further cognitive testing. Nonetheless, cognitive screening tests like the MMSE have the potential to help determine appropriate CI counseling, initially with expectations management, as well as potential adjustments to training approaches to improve performance, though further studies are needed to determine these changes. We do note however, that our participants all scored within the normal range on the MMSE (from 25 to 30), suggesting that this small range of MMSE performance in the normal range may not be meaningfully sensitive to differences in cognitive abilities. However, prior studies have demonstrated meaningful differences in patients scoring within the normal range of the MMSE. For example, Friedman et al (34) showed that older adult participants with MMSE scores from 25 to 30 showed meaningful differences on further cognitive testing using the Subtle Cognitive Impairment Test . Similarly, a study of adults with Parkinson’s with MMSE scores of 26 or greater showed meaningful ranges of cognitive performance on an extensive battery of neuropsychological testing (35). Thus, there is some evidence that MMSE scores within this normal range (25 to 30) may be meaningful. It should also be noted that we used a modified version of the MMSE that presents instructions and stimuli visually in addition to auditory, since audibility can impact performance on cognitive screening measures (36–38).

Prior studies have demonstrated the importance of inhibitory control, concentration, and lexical and phonological processing on CI performance (4, 11, 13, 39). The performance scores on these tasks (Stroop and TOWRE, respectively), however, were not correlated with very early CI performance in this study. There are not enough data in this study to claim there are differences in the importance of specific cognitive functions over time as patients adapt to and master CI use, especially with the lack of longer-term testing for this cohort. Despite this, our findings suggest temporal relevance of cognitive processing could be an interesting area of further research, especially with larger groups of CI users. Clinically, this may provide insight into the timing of specific auditory rehabilitation postimplantation, with the goal of improved peak performance.

In the current study, we saw no significant correlations between the preoperative cognitive test scores and the change in speech recognition scores from preoperative to postoperative 1- and 3-month testing. We were unable to assess the correlation with in-noise testing, given our sample size and a lack of preoperative testing in noise for most of our participants. We recognize the importance of accounting for preoperative performance, in addition to examining actual post-CI speech recognition scores, to take into consideration variability in preoperative hearing abilities. For example, 1 large-scale analysis examined the impact of cognition on improvement in speech recognition scores (40). Buchman et al (40), in a recent nonrandomized controlled trial, found no difference in mean change in speech recognition scores between patients with and without mild cognitive impairment. However, that study assessed cognition using the Montreal Cognitive Assessment (MoCA) alone. While effective at screening for mild cognitive impairment, the MoCA does not provide the detail of the cognitive domain obtained from a more comprehensive battery of cognitive testing. Further studies utilizing more detailed testing of particular domains of cognition, and possible effects on changes in speech recognition scores, is an important direction for future research.

## LIMITATIONS

A strength of this study is its prospective nature, which permits identifying preoperative abilities that might predict postoperative outcomes; however, the nature of our study design and small sample size create significant limitations. Our study is substantially limited by the size of our prospective cohort, and therefore, our findings should be interpreted with this in mind. Our participants were recruited just before and during the COVID-19 pandemic, which made further recruitment and retention challenging. With such a small sample, we were not able to account for confounding factors in our primary analyses, as well as heterogeneity in our participants. Specifically, our sample was not large enough to warrant multivariable analyses of our outcomes. Additionally, we were unable to extend our study to longer postoperative testing due to even fewer participants being able to return at 6 or 12 months for repeat testing. While we propose that our results are important and worth further investigation, it is difficult to extrapolate findings to the general CI population from such a small cohort. The nature of our particular cognitive and auditory tests limits the generalizability of our findings. Both types of tests provide a general picture of performance; however, these tests may not fully reflect real-world cognitive capacity or auditory function.

## CONCLUSIONS

Preoperative cognitive processing can help predict early outcome variability in adult CI users. We demonstrate that preoperative working memory, as tested by the forward digit span, appears to correlate with early CI performance on some speech measures at 1- and 3-month postoperative time points in our small cohort. Further investigation into cognitive processing and early CI performance, as well as how findings differ compared with longer-term performance is warranted. Ultimately, cognitive processing assessments could help customize counseling on performance and aid in determining appropriate auditory rehabilitation for CI recipients.

## FUNDING SOURCES

Development of measures and/or testing was supported by the American Otological Society Clinician-Scientist Award and the National Institutes of Health, National Institute on Deafness and Other Communication Disorders (NIDCD) Career Development Award 5K23DC015539-02 and R01DC019088 to A.C.M. Preparation of this manuscript was also supported by VENI Grant No. 275-89-035 from the Netherlands Organization for Scientific Research (NWO) to T.N.T.

## CONFLICT OF INTEREST STATEMENT

A.C.M. and T.N.T. have received grant funding support from Cochlear Americas for unrelated investigator-initiated research studies. A.C.M. has served as a paid consultant for Cochlear Americas and Advanced Bionics and is CMO and on the Board of Directors for Otologic Technologies. The other author has no conflicts of interest to disclose.

## DATA AVAILABILITY STATEMENT

The datasets generated during and/or analyzed during the current study are not publicly available, but are available from the corresponding author on reasonable request.
